# Targetoid ulcerating eruption in a previously healthy man

**DOI:** 10.1016/j.jdcr.2026.02.043

**Published:** 2026-03-02

**Authors:** Julia A. Giordano, Olivia G. Cohen, R. Hal Flowers, Margaret E. Moore

**Affiliations:** a Perelman School of Medicine, University of Pennsylvania, Philadelphia, Pennsylvania; b Department of Dermatology, University of Virginia, Charlottesville, Virginia; c Department of Pathology and Laboratory Medicine, University of Virginia, Charlottesville, Virginia

**Keywords:** AML, MPDMN, myeloid neoplasms, pDC proliferations, RUNX1

## Introduction

Mature plasmacytoid dendritic cell proliferations associated with myeloid neoplasms (MPDMN) are clonal expansions of plasmacytoid dendritic cells (pDCs) that occur in association with myeloid neoplasms including chronic myelomonocytic leukemia, acute myelogenous leukemia with pDC differentiation, myelodysplastic syndromes, and myeloproliferative neoplasms.^1^^,^^2^ MPDMNs typically manifest as nodules or aggregates of morphologically mature pDCs in the lymph nodes, skin, and/or bone marrow. This represents a case of MPDMN in which the cutaneous findings were a sentinel clinical sign of underlying acute myeloid leukemia (AML).

## Case presentation

**Fig 1:**
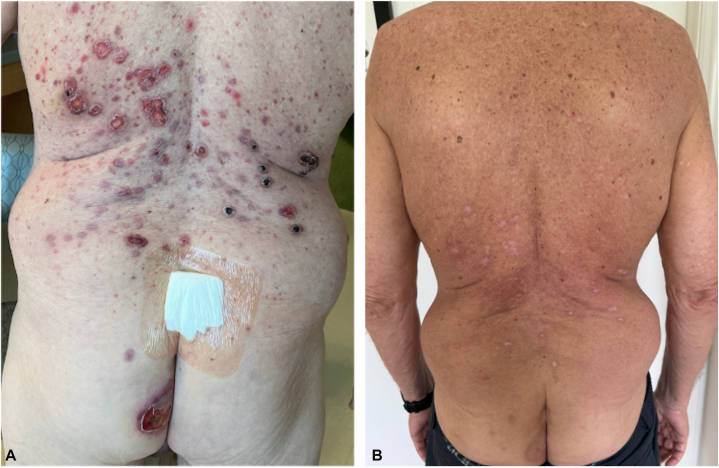
**A,** Edematous pink papules with central superficial ulceration and tender edematous violaceous purple targetoid papules and plaques with central eschar scattered on the trunk. **B,** Resolution of trunk lesions after treatment.

A 72-year-old previously healthy Caucasian, non-Hispanic male with a past medical history of hypothyroidism presented to the emergency room due to a 3-month history of a mildly pruritic and tender papular eruption on the trunk, which progressed to ulcerations and spread to his extremities, face, and oral mucosa (Fig 1, *A*).

According to the patient, the rash first appeared 3 months prior while in South Carolina and was initially diagnosed as chiggers and treated with clobetasol 0.05% for 1 week without improvement. Subsequent treatments from his primary care physician included 2 separate courses of oral doxycycline (100 mg twice daily for 1-2 weeks each) for presumed infection, followed by 2 courses of oral prednisone (40 mg daily with taper, 1-2 weeks each) for presumed inflammatory etiology. The rash was initially mildly itchy but progressed over several months to involve the face, trunk, buttocks, and upper legs. Over a rapid 2-week to 3-week period, the lesions became ulcerative and disseminated to involve nearly the entire body.

The patient presented to an outside hospital due to rapid progression of symptoms and was subsequently transferred to our institution for dermatologic and oncologic evaluation. He reported no preceding systemic symptoms, such as fever, night sweats, or unintentional weight loss. He reported no significant family history of malignancy. He has no known significant occupational exposures to any carcinogens. Physical examination demonstrated edematous pink papules with central superficial ulceration and tender edematous violaceous purple targetoid papules and plaques with central eschar scattered on the face, back, abdomen, buttocks, and upper thighs. Numerous painful oral erosions were present on the buccal mucosa and lateral tongue.

**Fig 2:**
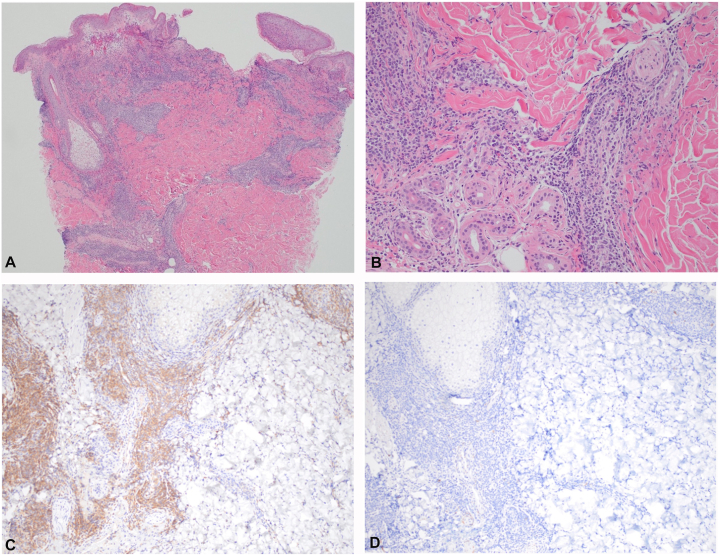
**A** and **B,** Histologic examination demonstrates a population of atypical monocytoid cells in a perivascular and periadnexal distribution (H&E, 40× and 200× magnification). **C,** Immunohistochemical stain for CD123 is strongly and diffusely positive in the cells of interest (100×). **D,** CD56 is negative (100×). *H&E*, Hematoxylin & eosin.

Upon admission to the hospital, complete blood count with differential was notable for normocytic anemia (hemoglobin 12.3 g/dL), thrombocytopenia (platelets 88 × 10^9^/L), absolute monocytosis (white blood cell count 9.1 × 10^9^/L, absolute monocyte count 2.8 × 10^9^/L), and rare circulating blasts. Skin punch biopsy was performed, followed by bone marrow biopsy with concurrent flow cytometry, next-generation sequencing, and karyotype analysis. Microscopic examination of the skin biopsy (hematoxylin & eosin) demonstrated an unremarkable epidermis, with a dermal population of medium-sized cells with a moderate amount of amphophilic cytoplasm, eccentrically placed nuclei, and moderately condensed chromatin, arranged in a perivascular and periadnexal distribution (Fig 2). By immunohistochemistry, the infiltrate was positive for Cluster of Differentiation (CD) 123, CD4, and T-Cell Factor 4 (TCF4). The cells of interest were negative for CD56, CD68, myeloperoxidase, muramidase, TDT, S100, CD1a, CD19, CD34, CD117, CD163, CD14, CD3, and CD20. Ki-67 showed a low proliferation index. Direct immunofluorescence was negative. Periodic acid–Schiff staining was negative for fungal organisms. Fungal cultures, as well as bacterial and acid-fast bacilli cultures, were also negative.

Bone marrow biopsy demonstrated a hypercellular marrow with increased myeloid blasts, consistent with AML. Flow cytometry concordantly showed an expanded immature myeloid population and increased pDCs. Next-generation sequencing analysis of the bone marrow detected pathogenic variants in *RUNX1*, *ASXL1*, *DNMT3A*, *FLT3* (TKD), and *IDH2*. Karyotype was normal (46, XY[20]). The constellation of features in the skin was compatible with a mature pDC proliferation, in the setting of an underlying myeloid neoplasm (AML).

The patient was initiated on chemotherapy (cladribine, low-dose cytarabine, and venetoclax) for AML. Treatment resulted in marked improvement of his cutaneous findings, with facial lesions resolving by day 3 and significant improvement of the trunk lesions by day 5 (Fig 1, *B*). The patient continued AML therapy and is currently in remission.

## Discussion

MPDMN are clonal pDC proliferations associated with myeloid neoplasia that remain a relatively newly recognized and undercharacterized entity within dermatology. MPDMN can be identified within the bone marrow and extramedullary sites such as lymph node, spleen, and skin. Within the skin, lesions can show a spectrum of appearances, including erythematous macules, papules, subcutaneous swelling, plaques, or vesicular eruptions, with or without pruritis.^1^^,^^3^ Lesions have been described in the literature in a variety of anatomic locations, including the trunk, face, and extremities, with no clear distribution pattern.^4^ Lymphadenopathy and splenomegaly may also be present.^3^ The condition is most common in elderly males, with 1 study describing a male to female ratio of 15:1.^1^^,^^3^ Diagnostic uncertainty based on physical examination findings alone is common, as reported by Dargent et al, stating that cutaneous pDC accumulations can mimic benign or inflammatory conditions,^3^ which was certainly the case in our patient who was initially incorrectly diagnosed with chiggers.

The differential for cutaneous pDC proliferations includes both hematologic and nonhematologic conditions. Clinically, lesions can mimic Sweet syndrome, lymphomatoid drug reactions, or lymphomatoid papulosis.^1^ Infectious etiologies, particularly deep fungal infections, can present with ulcerative papules but can be excluded by special stains and cultures. Other histologic mimics include non-pDC leukemias (leukemia cutis), lymphomas, Langerhans cell histiocytosis, and reactive inflammatory proliferations from drugs or autoimmune disease.^1^^,^^2^ A panel of immunohistochemical markers must be employed to establish a mature pDC phenotype in the infiltrate and exclude other hematologic malignancies.

Therefore, tissue biopsy is essential in the accurate identification of MPDMN. As in this case, histologic examination of skin lesions typically shows a proliferation of medium-sized cells with eccentric or ovoid nuclei, often arranged as nodular aggregates within the dermis and seen in a periadnexal or perivascular distribution. As described in Lee et al’s review, the cells of MPDMN demonstrate a pDC phenotype (CD4, CD123, CD303, CD304, and TCF4).^2^ In our case, immunohistochemical staining included CD123 and TCF4, both specific markers of pDCs, which supported the diagnosis. CD303 and CD304 were not performed, as these stains are not routinely available in our institutional laboratory. MPDMN cells lack the blastic morphology and CD56 expression characteristic of blastic pDC neoplasm.^5^^,^^6^ Ki-67 typically shows a low proliferation index.^2^ The cells of interest are typically negative for T-cell (ie, CD3), B-cell (CD20, CD19), histiocytic (CD68, CD163), and immaturity (TdT, CD34) markers by immunohistochemistry, aiding in exclusion of other hematologic disorders. While the genetic landscape of MPDMN is still being elucidated, pDCs appear to harbor the same mutational profile as the underlying leukemic clone, including myeloid blasts or abnormal monocytes.^7^ *RUNX1* mutations are highly characteristic of acute myelogenous leukemia with pDC differentiation,^5^ as seen in this case. One study suggests that this cohort of *RUNX1*-mutated AMLs has upregulation of a pDC transcriptional program, leading toward pDC differentiation and expansion.^8^ Because of their clonal relationship, treatment of the underlying myeloid neoplasm typically results in the improvement of cutaneous findings in MPDMN,^1^^,^^3^ as was the case with our patient. This is the first known documented case where cutaneous presentation preceded the diagnosis of the associated myeloid neoplasm, reinforcing the need for clinical suspicion and clinicopathologic correlation to avoid misdiagnosis.

Ultimately, MPDMN is a rare, although likely under-reported, condition associated with underlying myeloid malignancies. Diagnosis in skin biopsies can be challenging, particularly when a myeloid blast population or abnormal monocytic population is not localized in the same anatomic site. Rash resolution is expected with treatment of the underlying myeloid malignancy.

## Conflicts of interest

None disclosed.
